# Interpretable machine learning algorithms reveal gut microbiome features associated with atopic dermatitis

**DOI:** 10.3389/fimmu.2025.1528046

**Published:** 2025-05-01

**Authors:** Jingtai Ma, Yiting Fang, Shiqi Li, Lilian Zeng, Siyi Chen, Zhifeng Li, Guiyuan Ji, Xingfen Yang, Wei Wu

**Affiliations:** ^1^ National Medical Products Administration (NMPA) Key Laboratory for Safety Evaluation of Cosmetics, Guangdong Provincial Key Laboratory of Tropical Disease Research, School of Public Health, Southern Medical University, Guangzhou, China; ^2^ Guangdong Provincial Institute of Public Health, Guangdong Provincial Center for Disease Control and Prevention, Guangzhou, China; ^3^ Guangdong Provincial Center for Disease Control and Prevention, Guangzhou, China

**Keywords:** machine learning, random forest, light gradient boosting machine, extreme gradient boosting, SHAP value, partial dependence plot, interpretable machine learning

## Abstract

**Background:**

The “gut–skin axis” has been proposed to play an important role in the development and symptoms of atopic dermatitis. Therefore, we have constructed an interpretable machine learning framework to quantitatively screen key gut flora.

**Methods:**

The 16S rRNA dataset, after applying the centered log-ratio transformation, was analyzed using five different machine learning models: random forest, light gradient boosting machine, extreme gradient boosting, support vector machine with radial kernel, and logistic regression. Interpretable machine learning methods, such as SHAP values, were used to identify significant features associated with atopic dermatitis.

**Results:**

Random forest performed better than the other “tree” models in the validation partitions. The SHAP global dependency plot indicated that *Bifidobacterium* ranked as the strongest predictive factor across all prediction horizons, although the SHAP values for some features were still higher in support vector machine and logistic regression models. The SHAP partial dependency plot for “tree” models showed that the best segmentation point for *Bifidobacterium* was further from the origin compared to other features in the respective models, quantitatively reflecting differences in gut microbiota.

**Conclusion:**

Machine learning models combined with SHAP could be used to quantitatively screen key gut flora in atopic dermatitis patients, providing doctors with an intuitive understanding of 16S rRNA sequencing data to support precision medicine in care and recovery.

## Introduction

Atopic dermatitis (AD), alternatively referred to as eczema or atopic eczema, is one of the most prevalent inflammatory skin disorders encountered in the pediatric population, with its incidence increasing globally over the past few decades, affecting approximately 20% of children (1, 2). This condition is characterized by severe pruritus, which often leads to skin injury, significant sleep disruption, and a negative impact on overall quality of life (2).

Although the precise etiology of AD remains elusive, emerging evidence suggests that it results from a complex interaction between dysfunction of the epidermal barrier integrity, immune dysregulation, and the influence of environmental and infectious triggers (3–5).

This synergistic interaction elicits T-cell-mediated immune responses within the skin, including a predominantly T-helper 2 (Th2) cell response, which leads to the release of chemokines, proinflammatory cytokines, IgE production, and systemic inflammatory responses, giving rise to pruritic inflammation of the skin.

Recent studies have highlighted the pivotal role of intestinal flora development in facilitating optimal intestinal function and immunological development (6). The concept of the “gut–skin axis”, which emphasizes the reciprocal influence between gut flora and the skin, has emerged as a significant factor in the pathogenesis and manifestation of AD. The mechanisms underlying the gut-skin axis are multifaceted. First, metabolic pathways involving gut microbiota metabolites, such as short-chain fatty acids (SCFAs), play a crucial role. These metabolites enhance epithelial barrier function and reduce permeability (7, 8), while other metabolites contribute to the formation of a protective mucus layer (9). Secondly, gut microbiota plays a crucial role in the activation of both innate and adaptive immune mechanisms, collectively safeguarding the host and maintaining intestinal homeostasis. This includes modulating the differentiation of naive T cells to prevent excessive production of IgE and IgG4, as well as influencing interactions with Toll-like receptors (TLRs) and T-helper cell activity (10–15). Lastly, the “gut–brain–skin axis” further extends the interplay, linking microbiota modulation to stress-induced systemic and inflammatory skin disorders (16). The key neuromodulators involved in this axis are norepinephrine, serotonin, acetylcholine, and tryptophan (17).

However, a current challenge in this field is the complexity of 16S rRNA sequencing data, which poses difficulties in identifying key flora and the impact of intestinal flora quantitatively on the initiation and progression of AD. Addressing this challenge is crucial for advancing our understanding and treatment of AD.

Over the past few years, there has been a significant increase in the utilization of machine learning (ML) techniques in biomedical diagnosis and the identification of critical features. These techniques provide powerful tools capable of discerning intricate patterns and correlations within extensive datasets (18). ML algorithms have shown considerable utility across a range of clinical applications, including predicting disease outbreaks and personalizing treatment strategies (19, 20). Lundberg et al. (21) introduced an algorithm called “Shapley additive explanations (SHAP)”, a *post-hoc* interpretable algorithm that uses additive attribution to compute SHAP values, thereby enhancing the interpretability of previously opaque ML algorithms (22).

The main contributions of our study can be summarized as follows. We assessed the prediction performance of five different supervised ML algorithms including random forest (RF), Light Gradient Boosting Machine (LGBM), eXtreme Gradient Boosting (XGB), Support Vector Machine with the radial kernel (SVM), and logistic regression (LR) applied to analyze 16S rRNA sequencing data. To enhance classification accuracy and reduce overfitting, we adjusted the hyperparameters governing the sample weight distribution. Subsequently, we integrated ML algorithms with SHAP to develop an ML framework that enhances interpretability and identifies crucial features influencing the diagnosis of AD. This approach facilitates statistical and data-structure-related insights, contributing to an intuitive understanding of 16S rRNA sequencing data.

## Materials and methods

### Data collection

The dataset we analyzed and used to construct machine learning models was downloaded from the NCBI BioSample Database (https://www.ncbi.nlm.nih.gov/) with the accession number PRJNA501811. This BioProject is from a study conducted by Zhang et al. (23). The dataset contains 112 fecal samples, 43 from children with atopic dermatitis, and 69 from the healthy control group. To characterize the composition of the gut microbiome, the V4 hypervariable region of the 16S ribosomal RNA (rRNA) genes was amplified following DNA extraction from stool specimens.

Sequences were quality filtered, clustered into amplicon sequence variants (ASV) using the Unoise2 pipeline in USEARCH (https://drive5.com/usearch/) and taxonomically classified against the Silva-123 database (http://www.arb-silva.de/). To verify if the dataset reached a sufficient sequencing depth, alpha rarefaction analysis was performed using USEARCH, and classification was carried out up to the genus level.

### Taxonomic analysis

We analyzed the α-diversity (ACE and chao1 index) and β-diversity between the AD and HC groups. As for the β-diversity, both principal co-ordinates analysis (PCoA) based on the Bray–Curtis distance matrix and ADONIS test were employed to determine the significance of the difference.

To identify differentially abundant taxonomic features, linear discriminant analysis effect size (LEfSe) was applied via the “microeco” R package and “trans_diff$new()” function (24). The LEfSe bar graph plotted the significant difference in taxonomical features with a linear discriminant analysis (LDA) score of more than 2.0.

### Centered log-ratio

The centered log-ratio (CLR) transformation was initially applied to the abundance data pertaining to the designated bacterial genera. Given an observational vector comprising *D* “count” entities—such as sequencing reads or ASVs—in a given sample, denoted as *X* = [*X*_1_, *X*_2_, …, *X_D_*], the CLR transformation for that sample can be estimated by the following formula:


$$\mathrm{CLR}{\mathrm{(X}}_{i}\text{)=[}\ln(\frac{X_{1i}}{g(X_{i})}),\cdots ,\ln(\frac{X_{Di}}{g(X_{i})})]$$

Where *X_i_* is the list of features in a sample, *g*(*X_i_*) represents the geometric mean of the “count” vector *X_i_*, *X*_1_ *_i_* is the first feature in a sample, and $X_{D_{i}}$ is the last feature in a sample of *D* values.

The CLR transformation of the initial dataset requires replacing zero-count values to ensure accurate calculation, as the presence of zero-count values in the denominator of the CLR formula would make the computation infeasible. One approach to addressing this issue is to replace “0” counts with a value smaller than the detection limit (a count of 1). We followed the methodology proposed by Martín-Fernandez et al. (25), where we replaced each “0” count with 0.65 in our dataset, as our detection limit corresponds to a count of 1.

### Supervised ML modeling

Five kinds of different supervised ML algorithms were trained with the features of bacterial taxa using Scikit-learn: RF, LGBM, XGB, SVM, and LR.

The entire dataset was randomly divided into a training set (70%) and a testing set (30%), followed by hyperparameter optimization to enhance the performance of these algorithms. This process involved utilizing the GridSearchCV package (https://scikit-learn.org/stable/modules/generated/sklearn.model_selection.GridSearchCV.html) from Python 3 within the training set to tune the parameters through a 10-fold crossvalidation process. Importantly, during the hyperparameter tuning phase, the proposed models solely relied exclusively on the training set to obtain the optimized hyperparameters, ensuring that no information from the testing set was utilized. The specific optimized hyperparameters for each ML model are listed in **Supplementary Table S1**.

### Model evaluation

We obtained accuracy, precision, sensitivity, specificity, F1 score, and the receiver operating characteristic curve with area under the curve (AUC) by calculating the confusion matrix in Python 3. These metrics assess the ML models from several aspects.

### SHAP interpretability framework

ML was regarded as a black-box model since the impact of each feature was hard to assess on its prediction, especially in sequence analysis. In our study, the SHAP algorithm (21) treats each feature as a “contributor” to the outcome prediction and explains its significance in the particular prediction made by the ML model. The sum of the cumulative Shapley value for the specific prediction and the average prediction value provides the contribution of each feature for the individual. The contribution of each feature from the 16S rRNA data is given by the sum of the cumulative SHAP value for the particular prediction and the average prediction value.

In this manner, the SHAP value of each feature indicated how it influenced the prediction. Positive SHAP values (> 0) represent a positive effect on the predicted outcome, while negative SHAP values (< 0) manifest an adverse impact, showing a protective effect in our study. Global explanations calculate and rank the average SHAP values of each feature to visualize the importance of different features, while local explanations for “tree” models (XGB, LGBM, and RF) assess how SHAP values vary with abundance transformed by CLR and determine the best segmentation point by fitting the curve to analyze the features quantificationally.

### Statistical analysis

The statistical analysis in this study was performed using R software (version 4.2.0) and Python 3. Continuous variables were reported as mean ± standard deviation for normally distributed data and as median (interquartile range) for skewed variables. Two-sample *t*-tests or Wilcoxon’s rank sum tests were utilized to draw statistical inferences. Prior to building the ML models, random sampling was employed to split the dataset into training (70%) and test (30%) sets, respectively. The level of significance was set at *p<* 0.05.

## Result

### Overview of the 16S rRNA gene sequencing data

According to phylogenetic taxonomic levels, we performed 16S rRNA gene sequencing of all stool samples (*n* = 112) with AD groups (*n* = 43) and health control (HC) group (*n* = 69), and a total of 9,155,243 sequences were sorted into 2,811 ASVs.

The Shannon rarefaction curve was employed to verify whether the dataset reached sufficient sequencing depth. As shown in **Supplementary Figure S1**, with the increasing sequencing volume, the number of species did not increase significantly, indicating that the sample size was sufficient for our study and that the sequencing depth met the demands of the subsequent data analysis.

### Richness and diversity of gut microbiota in ADs

**Figure 1:**
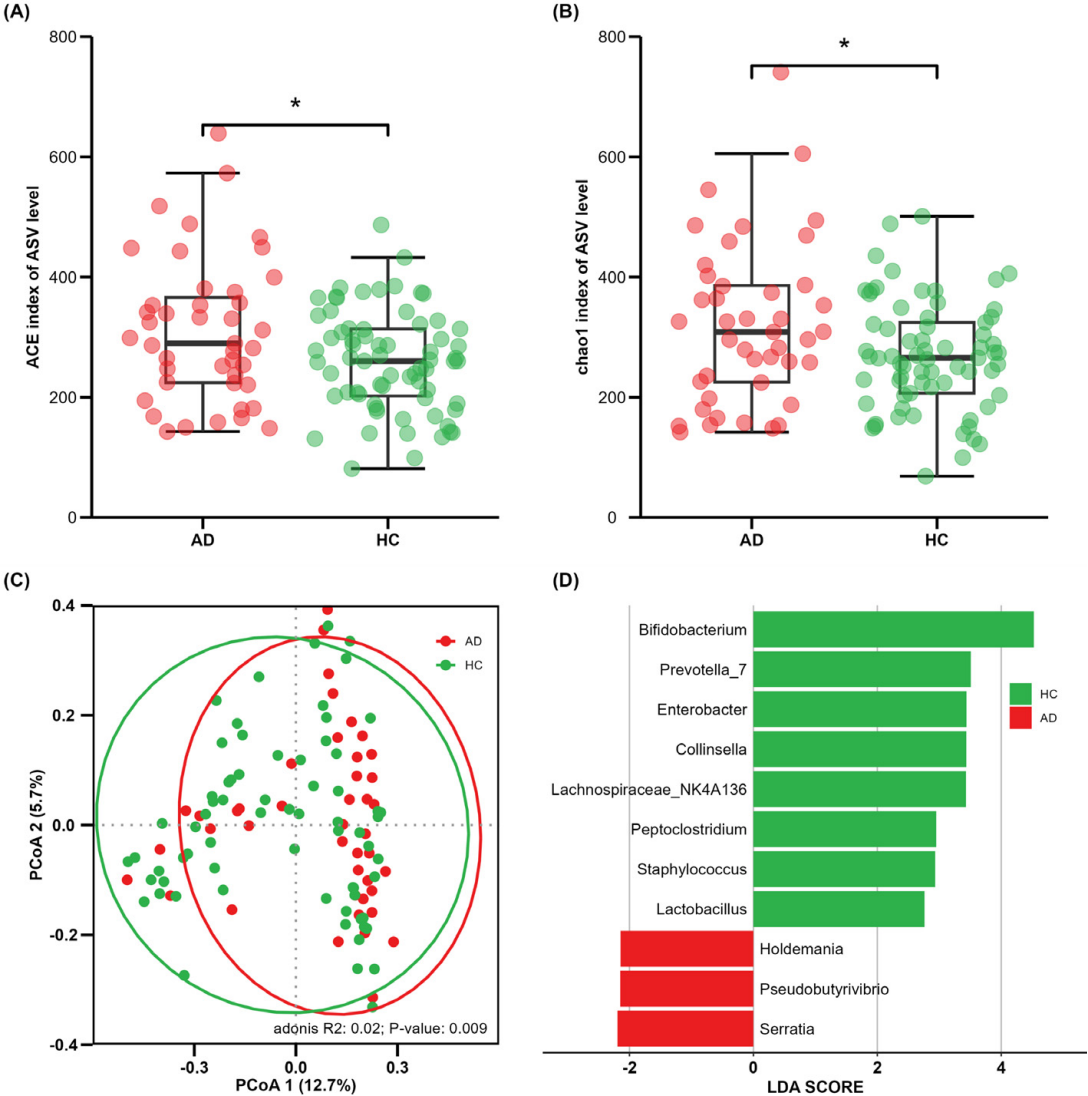
Comparison of alpha and beta diversity between AD and HC groups. Each point in the figure represents a sample. **(A)** ACE index; **(B)** Chao1 index; **(C)** principal co-ordinates analysis, where shorter distances between samples indicate greater similarity in species composition; **(D)** cladogram of linear discriminant analysis (LDA) effect size (LEfSe) analysis of microbial abundance. *P<0.05.

AD samples (*n* = 43) demonstrated increased gut microbiota richness and diversity compared with health control (HC) samples (*n* = 69), as measured by the Ace index (*p* = 0.019) and chao1 index (*p* = 0.028) (**Figures 1A, B**), respectively. Beta diversity was applied to assess the structural composition similarity of gut flora. As shown in **Figure 1C**, PCoA analysis based on the Bray–Curtis distance revealed that Co-ordinate 1 accounted for 12.7% and Co-ordinate 2 occupied 5.7%. ADONIS demonstrated significant differences between the AD and HC groups (*R* ^2^ = 0.02, *p* = 0.009) (**Figure 1C**).

In the LEfSe results, significant differences in microbial proportion were observed between the AD and HC groups. Eight genera were overrepresented in HCs, and three genera were overrepresented in ADs at the genus level (**Figure 1D**). *Bifidobacterium* showed the most significant differences, with the LDA score greater than 4. These findings suggest that there is gut microbial dysbiosis in AD patients, and different states have their own unique characteristics.

### Model evaluation

A gut microbiota-based signature transformed by CLR can be used for predicting atopic dermatitis. Based on the comprehensive 16S rRNA analysis above, we next assessed the performance of gut microbiota as biomarkers using ML models, including XGB, LGBM, RF, SVM, and LR. In this study, model performance evaluation was calculated including accuracy, recall, precision, sensitivity, specificity, F1 score, and AUROC. As shown in **Table 1**, the algorithms based on “tree” models performed better than SVM and LR, demonstrating the high performance of the models. The RF performed better than the other “tree” models in the test set. The LGBM also performed well except for precision (85.70%) and specificity (81.30%), which were lower than RF’s 100.00% and 100.00%, respectively. The accuracy and specificity of the XGB model were higher than those of the LGBM model, meanwhile, the sensitivity and recall were lower than those of the other two models based on the decision tree classifier. These findings indicated that according to different statuses, we can choose different models.

**Table 1:** Model performance of the proposed ML models on the testing set.

Classifier	Accuracy	Precision	Sensitivity	Specificity	F1 score	AUROC
RF	94.10%	100.00%	91.30%	100.00%	95.45%	0.9817
LGBM	91.20%	85.70%	100.00%	81.30%	92.30%	0.9817
XGB	91.20%	95.20%	90.90%	91.70%	93.00%	0.9780
SVM	85.30%	90.50%	86.40%	83.30%	88.40%	0.8370
LR	82.40%	85.70%	85.70%	76.90%	85.70%	0.9194

### SHAP interpretability framework

**Figure 2:**
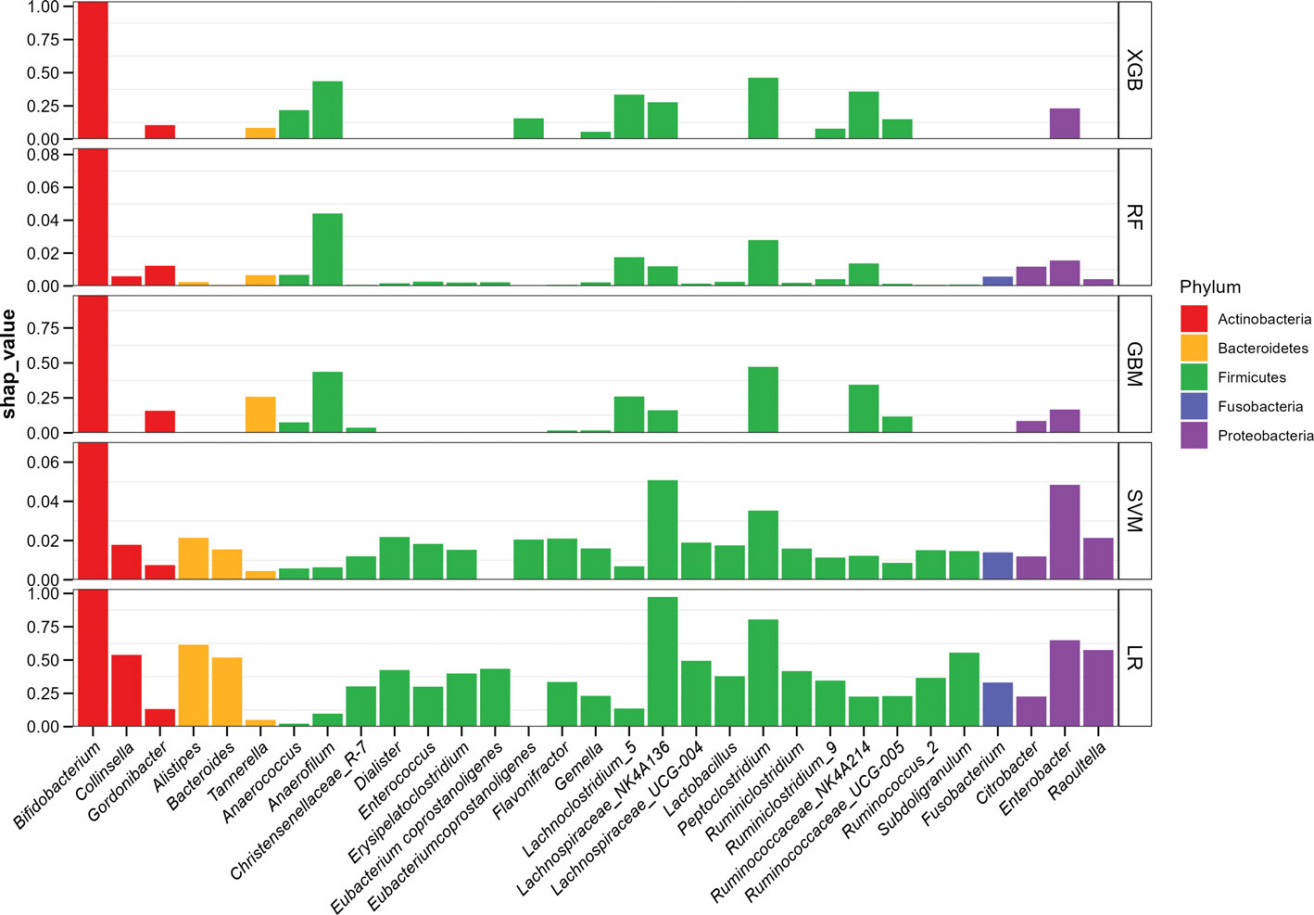
ML-SHAP global dependency plot analysis showing the distribution of feature importance in AD occurrence risk.

In consideration of the “black box” dilemma, the SHAP algorithm was conducted to visually exhibit each feature’s importance to the AD predicted by the ML models. The SHAP global dependency plot ranks the SHAP value of the features, as shown in **Figure 2**, including the top 30 significant features most correlated with the outcome in descending order. The vast majority of bacterial genera belong to four dominant phyla, and *Bifidobacterium* ranked as the strongest predictive factor for all prediction horizons, playing a decisive role in all models, though the SHAP values of some features were still higher in the SVM and LR models.

**Figure 3:**
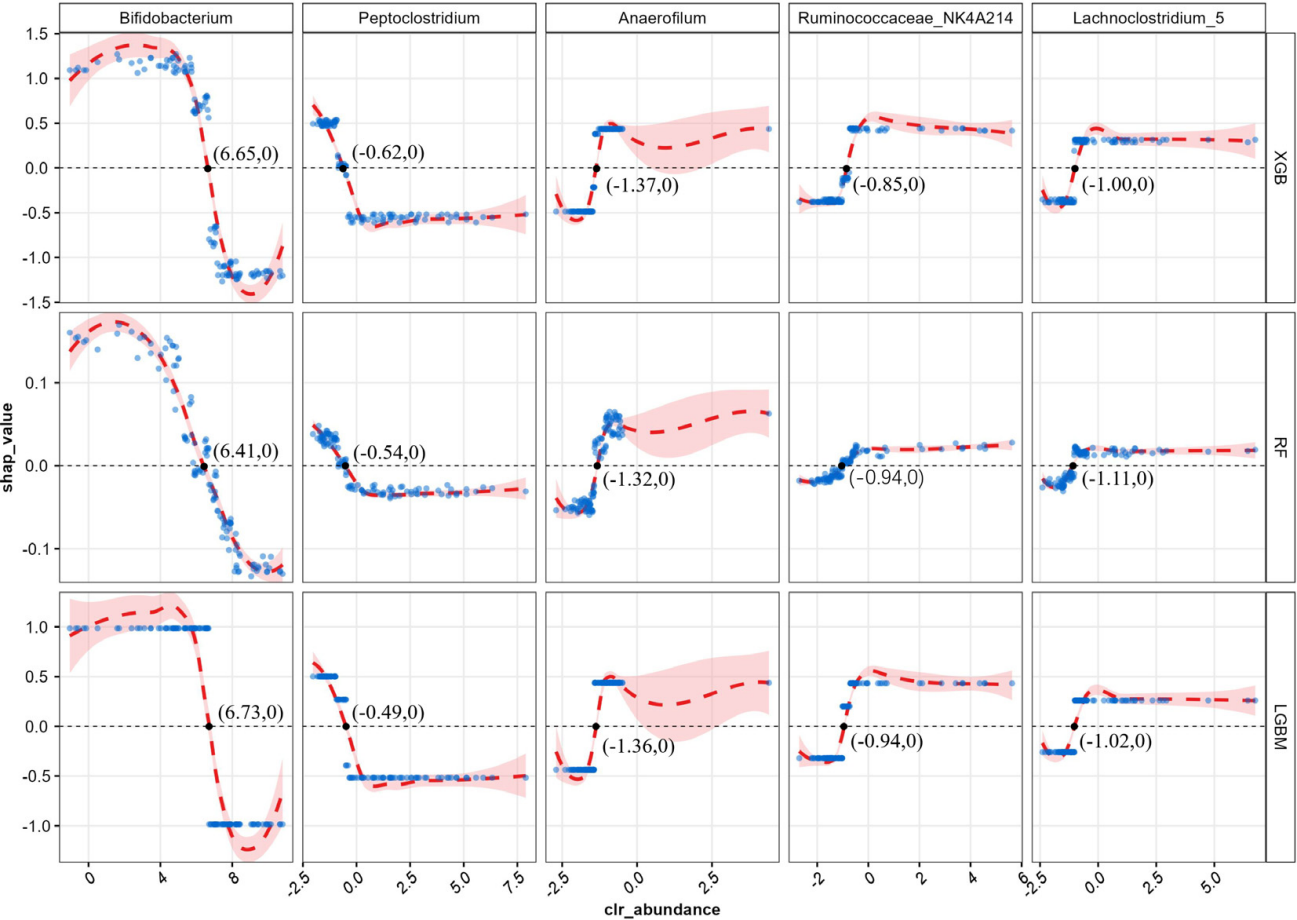
ML-SHAP partial dependence plot analysis showing the distribution of feature importance for AD occurrence risk.

However, the SHAP global dependency plot showed limitations in depicting the association between each feature and its SHAP value visually. Consequently, to gain a deeper understanding, we drew the SHAP partial dependency plot for “tree” models, as depicted in **Figure 3**. The performance of the SVM and LR fell flat, so we did not take the two models into account. In the plot, each point represents an abundance and its SHAP value, with the abscissa indicating the magnitude of the transformed feature value and the ordinate showing corresponding SHAP values.

We noted that different models provided multiple interpretations of 16S rRNA data. A specific predictive behavior was exhibited in the XGB model, which predicted a designated threshold to detect the positive and negative relationships of the predictors with the outcome result, and the greater the distance, the stronger the effect. The LGBM model showed a clear dose–response relationship and the RF indicated more smoothly. The best segmentation point falls on 6.65(XGB), 6.73(LGBM), and 6.41(RF), as for *Bifidobacterium*, the intersection points between the asymptote and line where y = 0, which are greater than other features in respective models, quantitatively reflecting the CLR-abundance of gut microbiota in AD and HC group.

## Discussion

This study aimed to explore and discuss the significantly dysregulated microbiota in the AD group compared to the HC group, using ML algorithms combined with SHAP techniques to visualize features and their respective weights. Through 16S rRNA analysis, we found significant differences in both α- and β-diversity between the AD and NC groups. Five types of machine learning algorithms, combined with the SHAP algorithm, were used to identify significantly dysregulated microbial taxa.

Statistical methods used in bioinformatics and model construction consistently indicated that *Bifidobacterium*, an intestinal probiotic, was the strongest predictive factor. Previous studies have shown that *Bifidobacterium* is enriched in healthy infants (26, 27) and markedly reduced in the gut of infants with AD (28), as well as in those with other atopic infantile diseases (27).

The pathogenesis of AD involves multiple mechanisms, including a reduction in regulatory T cells (Tregs), which can impair immune responses and lead to an imbalance in the activation of TH1/TH2 cytokines (29). Recent evidence has increasingly highlighted the vital role of intestinal microbiota in regulating the immune system (6). Alterations in the microbiome can impact both host immunity and its response to antigens, thereby contributing to the development of allergies. *Bifidobacterium* has been proven to induce a regulatory dendritic cell (DC) phenotype that enhances the induction of Tregs (30, 31). Patients lacking *Bifidobacterium* tend to exhibit a more proinflammatory Th2/Th17 profile, whereas those with abundant *Bifidobacterium* display a more anti-inflammatory profile (32). Furthermore, early colonization by *Bifidobacterium* modulates B-cell responses, with infants colonized early showing higher levels of memory B cells at 4 and 18 months (33), and increased salivary secretory IgA at 6 months (34). These findings suggest that the presence of *Bifidobacterium* promotes B-cell activation, maturation, and ultimately antibody production, thereby limiting immune activation and sensitization.

The synthesis and secretion of microbial metabolites constitute a pivotal mechanism through which the gut microbiota exerts its modulatory effects on immune function, thereby exerting a significant impact on overall health outcomes. SCFAs, one of the microbial metabolites, mainly produced by *Bifidobacterium* (35), are by-products of bacterial fermentation and abundant microbial metabolites present in the colon. Low fecal concentrations of these SCFAs (such as propionate, butyrate, and acetate) have been associated with the occurrence and development of allergic diseases (e.g., atopic dermatitis, food allergy, asthma, allergic rhinitis) (36, 37). In addition, SCFAs might potentially interact with mesenchymal stem cells (MSCs) through G protein-coupled receptors (GPCRs), modifying their differentiation potential through the inhibition of histone deacetylase (HDAC) activity, thereby inducing distinct infant immune responses (38). Furthermore, evidence suggests that SCFAs enhance intestinal epithelial integrity and regulate various immune cell populations, including dendritic cell maturation, Treg differentiation, and antibody production (39).

Previous studies have shown that the proportion of *Peptoclostridium* is more likely to relate to intestinal health and recovery from imbalances (40). *Anaerofilum* (41, 42) and the genus *Lachnoclostridium* (43, 44) were associated with an increased risk of immune diseases, consistent with the trend in **Figure 3**: the higher the abundance, the higher the likelihood of having atopic dermatitis. In addition, there is a correlation between the *Ruminococcaceae_NK4A214_group* and SCFAs (45).

As for model evaluation, the “tree” models had greater performance. *Bifidobacterium* ranked as the strongest predictive factor for all prediction horizons and exclusively demonstrated the decisive role in all models. Perhaps due to interference from other features, SVM and LR had lower efficiency in forecasting. In contrast, the “tree” models, which may eliminate interference, manifested as a decrease in the weight of other features. RF showed the best performance, which was similar to the findings of other studies (46, 47). LGBM and XGB, both of which learned from the mistakes of previous models using boosting techniques, also showed good performance in other fields (48, 49).

It is always a challenge to correctly interpret the great predicted performances and understand why they perform well, especially in ML’s black-box tree integration model. Therefore, we applied the SHAP algorithm to evaluate the importance of features in all possible combinations of the permutations and visualize them in every prediction model, helping us provide valuable insights into the most influential features (50). To visually represent the relationship, SHAP partial dependency plots were constructed, which can exhibit how the “tree” models presorted the features according to the numerical value, followed by the fitted curve. We can quantitatively observe the relationship between abundance (or the position of abundance proportion distribution) and SHAP value.

The SHAP value in all “tree models indicates that as the proportion increases, the influence shifts from promoting to preventing, or vice versa, at a certain point. This suggests that there may be a threshold for gut microbial dysbiosis associated with AD, with different features having different thresholds. Since the CLR used a zero-average matrix data approach, the segmentation point, located at the intersection between the fitted curve and the *y*-axis, can be defined as a quantitative reference point for the relationship between different features and the outcome. As for *Bifidobacterium*, its critical value is higher than that of other genera, indicating that in the HC group, its anti-eczema effect can only be exerted when *Bifidobacterium* becomes dominant and its relative abundance exceeds the threshold. In contrast, in the AD group, the homeostasis of the intestinal flora appears to be disrupted. On the contrary, *Ruminococcaceae_NK4A214_group*, a member of the *Ruminococcaceae* family, showed an opposite trend to *Bifidobacterium*, with increased abundance reported in children who have or are likely to develop AD (28). According to the CLR formula, CLR values can be converted back to abundance values using the equation: Abundance = *e*^^CLR value^*g(Xi), where *g*(Xi) is the geometric mean of the feature in a sample. The reference values corresponding to the segmentation points—6.65(XGB), 6.73(LGBM), and 6.41(RF)—are 777.78, 607.89, and 837.15, respectively. This indicates that in order to exert bifidobacterium’s protective effect, its abundance must be restored to approximately 600 to 800 times the geometric mean. In contrast, the reference values for the other mentioned genera ranged from 1.2 to 1.8 times the geometric mean, which may serve as a reference for assessing the restoration of intestinal flora homeostasis.

In summary, the SHAP algorithm can visually explain which specific characteristics related to gut microbial dysbiosis are associated with a higher (or lower) risk of AD. While machine learning algorithms have the potential to assist medical researchers in clinical and mechanistic studies, their ambiguous processes and high predictive performance create a “black box” dilemma. To address this, noninvasive gut microbiota data were analyzed, and ML algorithms combined with SHAP were used to visualize the influence of each relevant feature on the model output. This approach, supplemented by medical knowledge, offers new perspectives and profound insights for the subsequent exploration of mechanisms and practical therapeutic targets.

This study also has several limitations. The hospital-based sample size was not large enough to cover all aspects of the pathogenic mechanism in gut microbiota, and other confounders have not yet been considered, which may limit the generalizability of our findings. Future research will expand the study to include different hospitals and regions, as well as other confounders, to verify the reliability of our results.

## Conclusions

The results of our study indicate that the machine learning models combined with SHAP feature attribution analysis could be used to screen key gut flora, such as *Bifidobacterium*, and quantify their relationships. Prospective trials are needed to validate these findings and further refine the understanding of association and causality. For researchers and clinicians, interpretable machine learning algorithms are valuable tools for extracting insights and making accurate predictions from 16S rRNA sequencing data to support precision medicine in patient care and recovery.

## Data Availability

The datasets presented in this study can be found in online repositories. The names of the repository/repositories and accession number(s) can be found in the article/**Supplementary Material**.
