# Supplementary figures and images for: Interpretable machine learning algorithms reveal gut microbiome features associated with atopic dermatitis
Source: Front Immunol. 2025 May 1;16:1528046. doi: 10.3389/fimmu.2025.1528046 (PMC12078218; doi:10.3389/fimmu.2025.1528046)

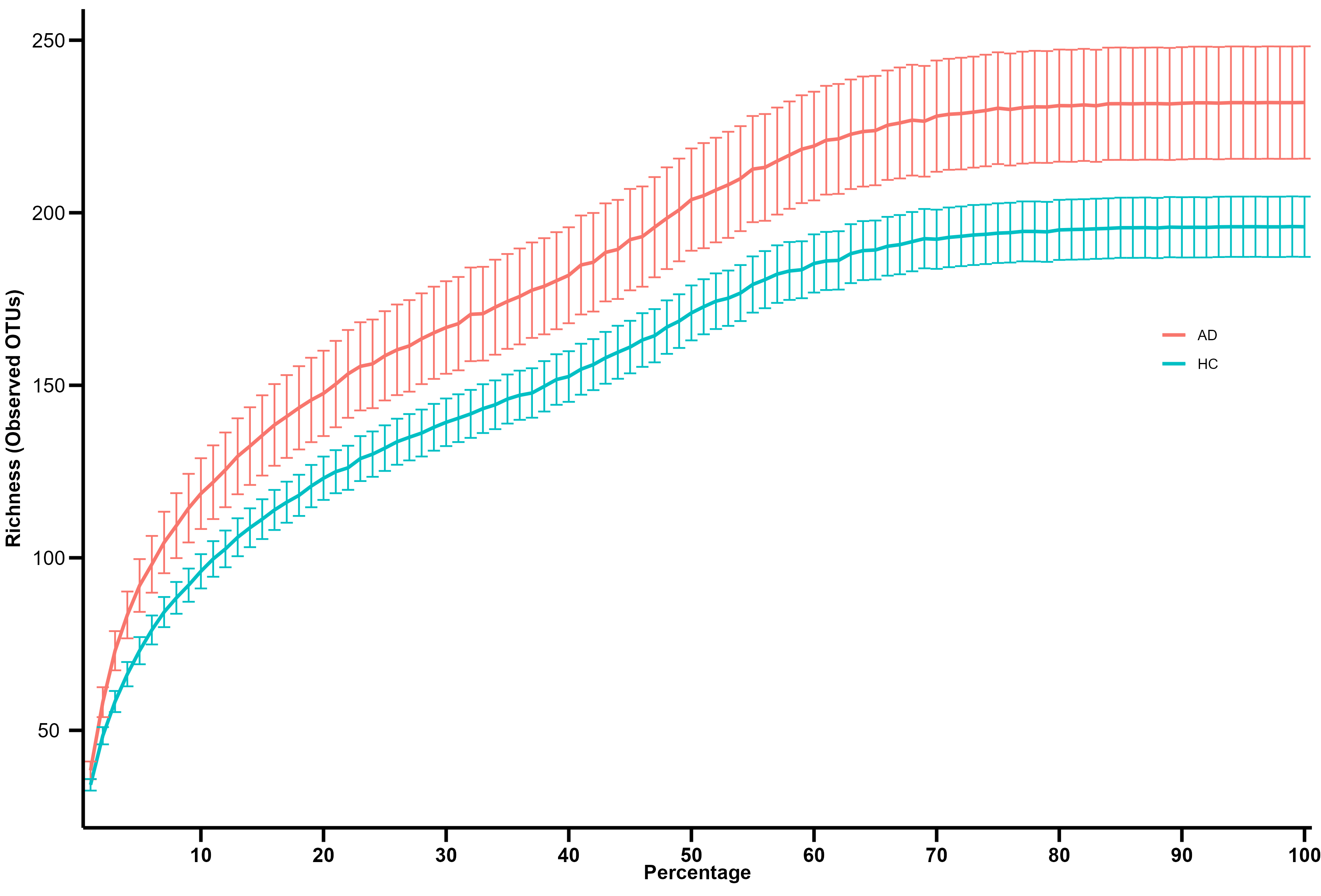

Supplement: Supplementary file 2 [file Image1.tiff]
